# A Microwave Digestion Technique for the Analysis of Rare Earth Elements, Thorium and Uranium in Geochemical Certified Reference Materials and Soils by Inductively Coupled Plasma Mass Spectrometry

**DOI:** 10.3390/molecules25215178

**Published:** 2020-11-06

**Authors:** Sharayu Kasar, Rajamanickam Murugan, Hideki Arae, Tatsuo Aono, Sarata Kumar Sahoo

**Affiliations:** Environmental Radionuclides Research Group, National Institutes for Quantum and Radiological Sciences and Technology (QST), Chiba 263-8555, Japan; kasar.sharayu@qst.go.jp (S.K.); murugan.rajamanickam@qst.go.jp (R.M.); arae.hideki@qst.go.jp (H.A.); aono.tatsuo@qst.go.jp (T.A.)

**Keywords:** JSd-2, chemical digestion, rare earth elements, Th, U, ICP-MS

## Abstract

Two different digestion methods—microwave digestion (Mw) and Savillex digestion (Sx)—were used to evaluate the best quality control for analysis of the rare earth elements, Th and U in the geochemical certified reference material JSd-2, supplied by the Geological Survey of Japan (GSJ). The analysis of trace elements was carried out using inductively coupled plasma mass spectrometry (ICP-MS). The digestion recovery was > 90% for almost all elements by both methods. Mw-4 (four repeats of the microwave digestion) was found to be more effective and faster than Sx. In order to evaluate the efficiency of Mw-4, three other GSJ certified reference materials, JLk-1, JB-1 and JB-3, as well as five different soil samples from Belarus, Japan, Serbia and Ukraine were also analyzed. The Mw-4 method was seen to be promising for complete digestion and recovery of most of the elements. The U/Th ratio showed some heterogeneity for Ukraine and Serbia soils affected by Chernobyl nuclear power plant accident and depleted uranium contamination, respectively. This method can be successfully applied to any type of soils for elemental analyses.

## 1. Introduction

Concerns over accumulation of trace elements, rare earth elements (REEs), uranium (U) and thorium (Th) and the possible ecological effects their accumulation has on the environment are increasing due to rapid industrialization. The REEs are extensively used in metallurgy (for metal refining and metal alloying), the automotive and petrochemical industries (for catalysts), manufacturing (for coloring of glass and ceramics), phosphors (for LEDs, compact fluorescent lamps and flat panel displays), lasers, rechargeable solid-state batteries and fiber optics. Naturally occurring minerals like monazites and many other accessory minerals are one of the principal sources of REEs and Th. The primordial radioactive elements U and Th are ubiquitously present in the earth’s crust. Various agencies or committees such as the World Health Organization (WHO) [1], the United Nations Scientific Committee on the Effects of Atomic Radiation (UNSCEAR) [2], the Ministry of Education, Culture, Sports, Science and Technology (MEXT) [3] and the United Nations Environment Programme (UNEP) [4] have introduced generic guideline values relating to the maximum levels of contaminants that can be released into or be contained in the environment. Enriched uranium in the form of mixed oxide was used as fuel in nuclear reactors like the Chernobyl Nuclear Power Plant (CNPP) and the Fukushima Daiichi Nuclear Power Station (FDNPS). Nuclear accidents at these plants released various radionuclides (^90^Sr, ^134^Cs, ^137^Cs, ^131^I, ^127m^Te, ^129m^Te, ^141^Ce, ^144^Ce, ^143^Pr, ^147^Nd, ^236^U etc.) into the environment [5]. During the Kosovo conflict, depleted uranium weapons were fired causing contamination in the many regions [6]. To estimate any changes in their concentration levels due to natural or anthropogenic sources, it is essential to monitor these elements in the environment particularly in soils. Therefore, analysis of soils contaminated by the CNPP and FDNPS accidents or any other nuclear event, for elements including REEs, Th and U is important from a radioecology viewpoint.

Analytical techniques e.g., instrumental neutron activation analysis [7], inductively coupled plasma-atomic emission spectrometry [8], inductively coupled plasma mass spectrometry (ICP-MS) [9,10] and laser ablation ICP-MS (LA-ICP-MS) [11,12] are used to measure multi-elements from environmental samples. ICP-MS is a powerful and rapid technique with high sensitivity and a low detection limit (ng/L). It requires only a small sample volume for rapid analyses. For this purpose, soils need to be brought to a solution form via a soil digestion technique. Soil digestion techniques include microwave digestion (Mw), leaching using acid attacks, block digesters, bomb method, soil fusion with a flux and high pressure asher digestion. In a comparison with other digestion techniques, block digester methods are ideal for total nitrogen and phosphorous digestion than other multi-elements. Microwave digestions ensure safety and better throughput than block digester methods [13]. Problems with maintenance of bombs restrict their risk-free use and that is also the situation for the high pressure asher. When there are just a few target elements, fusion with a flux is suitable. The high concentration of total dissolved solids in fusion using flux is troublesome during purification of trace elements especially for uranium isotope ratio measurements using thermal ionization mass spectrometry. For multi-elemental analyses, impurities due to the flux cause difficulty in the blank measurement [14]. Hence, leaching using acid attacks and microwave digestion techniques are preferable.

Quality control of digestion is based on the analysis of a certified reference material using a well-tested analytical method and comparison of the results obtained with the certified values [15]. First, two different types of digestion methods viz. Mw and leaching using acid attacks in Savillex bottles (Sx) have been applied in the present work on the geochemical certified reference material (CRM), JSd-2, supplied by the Geological Survey of Japan (GSJ). A total of 18 trace elements (La, Ce, Pr, Nd, Sm, Eu, Gd, Tb, Dy, Ho, Er, Tm, Yb, Lu, Cs, Sr, Th and U) were analyzed using ICP-MS for better comparison between the two digestive methods. An assessment of the best quality control digestion technique was carried out by analysis of the CRMs, JSd-2, JLk-1, JB-1 and JB-3. Second, the same method has been applied for REEs, Th and U analyses in five different soils: two contaminated by the CNPP accident, one collected from Ukraine and the other from Belarus; two collected from Japan, one contaminated by the FDNPS accident and another from Hiroshima; and the final one contaminated by depleted uranium weapons, collected from Serbia in the present work. Such type of approach is scarcely available in literature.

## 2. Results and Discussion

### 2.1. Comparison between Digestion Methods for JSd-2

The selection of a reference material is dependent on abundance of the elements of interest in that material and the soils to be examined. It has been observed that Fukushima soils are richer in Al_2_O_3_ and Fe_2_O_3_ oxides than Chernobyl soils [16,17]. The certified values of JSd-2 (Japanese sediment) show high content of both these oxides which is similar to the trend of Fukushima soils [18]. The concentrations of REEs, Th and U in JSd-2 are in the range of reported values in Japanese soils [19,20]. Hence, JSd-2 was selected as a reference material in the present work, being a representative to Japanese soils, to evaluate effective digestion method. The application of this method was tested for the digestion of the soils contaminated due to nuclear events to analyze multi-elements using ICP-MS. 

**Figure 1:**
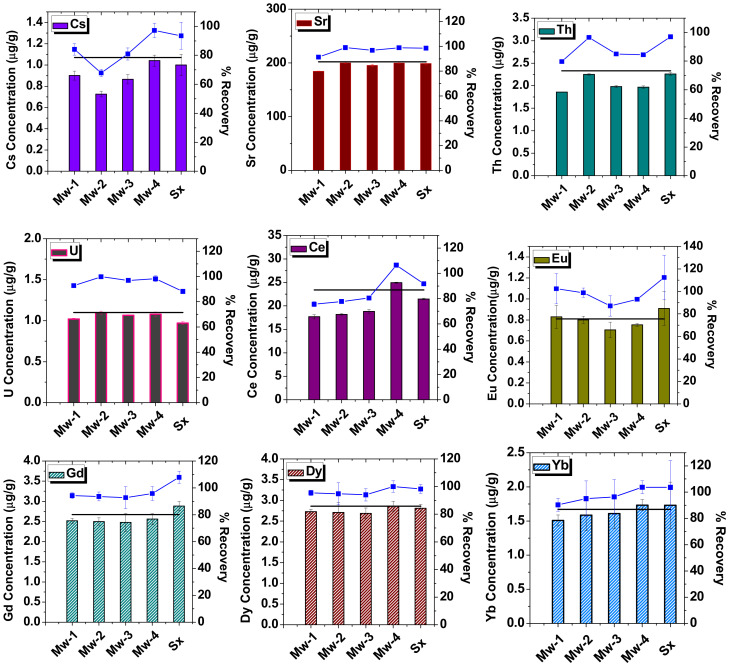
Comparison between microwave digestion (Mw) and Savillex digestion (Sx) for JSd-2; concentration (μg/g) and recovery (%) of Cs, Sr, Th, U, Ce, Eu, Gd, Dy and Yb obtained after digestion (The solid line corresponds to the certified value of the respective metal. Errors in the method of analysis are represented as SD. *n* = 3.).

Concentrations (in ppm or μg/g) of trace elements such as Cs, Sr, Th, U, Ce, Eu, Gd, Dy and Yb analyzed after different digestion methods for JSd-2 are given in Figure 1. Errors in the method of analysis are represented as standard deviation (SD) which refers to the precision of method. Most of the elements were not recovered (<80%) after Mw-1 or Mw-2 (the numbers indicate the times a microwave digestion was done). Out of 18 elements (La, Ce, Pr, Nd, Sm, Eu, Gd, Tb, Dy, Ho, Er, Tm, Yb, Lu, Th, Sr, Cs and U) only seven (Sr, Th, U, Eu, Gd, Dy and Yb) were recovered successfully (>90%), after Mw-3 as shown in Figure 1, whereas the other elements were recovered at ~80%. Digestion recovery of all 18 elements was found to be >90% after Mw-4 for JSd-2 (Table 1) [18] and a clear solution was observed. Thus, the Mw-4 method was found to be the most efficient one amongst different microwave digestions in this work. It took about 9 h to finish all four digestions. For Sx, the recovery of most of the elements was promising (about 80% or more) (Figure 1) and the solutions were almost clear. Depending on the hot plate size, it would be possible to digest more than 20 samples simultaneously. But there could be sample loss and cross contamination while opening or closing many Savillex vials. Also, the method was time consuming, taking about 48 h. It has been reported that, in conventional microwave digestions when samples are not completely digested, an added open digestion step is needed to ensure complete digestion, which is time consuming [13]. However, the Mw-4 method did not require additional open digestion step. This corroborates the finding of the present work that the Mw-4 method is a better choice than Sx method for simultaneous multi-elemental and rapid analysis using ICP-MS in ~1–2 g of soil samples.

### 2.2. Rare Earth Elements and Selected Elements in Certified Reference Materials

Table 1 summarizes the analytical results obtained by ICP-MS for the CRMs that had been prepared using the Mw-4 method. The table also lists their certified values from the literature [18]. The ratio (%) of the mean measured values to the certified values is >90% for all elements and expressed as accuracy with analytical recovery. The accuracy as relative bias (RB%) of the measurement is <10%. All the results are reported with their experimental standard uncertainty. The relative standard deviation (RSD%) is <10% for all analyzed elements. This states that, the reproducibility as a precision of the method is in good agreement with the certified values of all elements. The accuracy and precision were combined to estimate the combined standard uncertainties (u) for the different elements [21,22]. To estimate expanded uncertainty (U_AD_) the ISO Guide to the Expression of Uncertainty in Measurement (GUM) was followed [23]. It was observed that, the absolute difference (AD) between the mean measured value and the certified value [18] is less than twice the uncertainty of absolute difference or the estimated expanded uncertainty (U_AD_). This supports the good performance of the presented method for sample digestion since there is no significant difference between the measured and the certified values and they are within the 95% confidence level. Overall, the estimated uncertainties obtained by combining the accuracy and precision (u) of these CRMs are found to be comparable to those calculated by GUM methodology (U_AD_). Major and trace elements have been determined in a variety of geochemical CRMs such as JSd-2, JLk-1, JB-1 and JB-3 using LA-ICP-MS [11]. However, the recovery for some of the elements deviated from certified values (RB% > 20%). Similar results have been observed in analysis of GSJ standards after carbonate fusion and acid digestion for precise determination of rare earth elements using ICP-MS [24]. Therefore, the aforementioned methods reported in literatures are not suitable for rapid and precise analysis of element concentrations in soils [11,24]. On the other hand, Mw-4 is an efficient rapid digestion method as explained in the present work and can be applied effectively to soil, sediment as well as rock samples of similar origin to JSd-2, JLk-1, JB-1 or JB-3.

**Figure 2:**
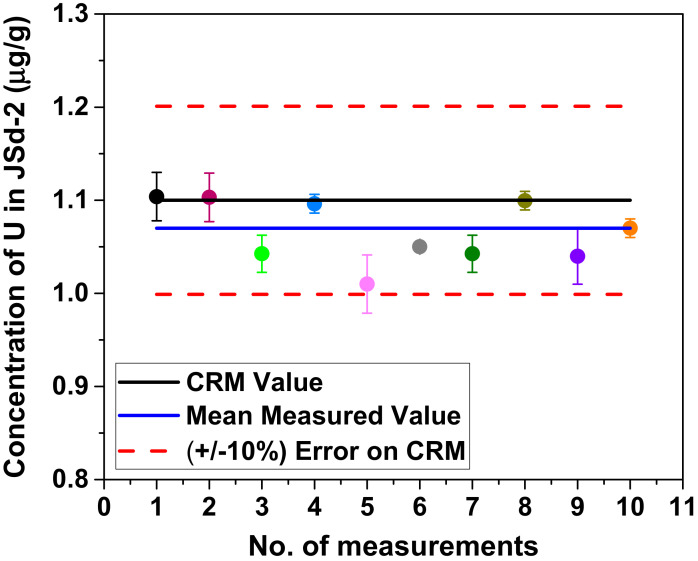
Measured recovery on 10 days for U in JSd-2 prepared by the Mw-4 method. (Errors in the method of analysis are represented as SD).

In order to estimate the long-term analytical reproducibility of the Mw-4 method, JSd-2 digestion was carried out on 10 days. The measured recovery results for U are plotted in Figure 2. 

The precision (RSD%) on the mean measured value from this analysis is ~3%. All measurements are not only within (± 2SD) of the mean measured value but also within (± 10%) of the certified value [18]. The absolute difference between the mean measured value and the certified value is less than twice the uncertainty of the absolute difference, confirming the good performance of the presented method. As there is no significant difference between these values, the method is confirmed to be accurate and precise. Since uranium oxide is used as a fuel in many reactors, determination of U concentration in soils will lead to understand its effects on the environment if nuclear fuel materials are released by an accident [5]. Therefore, only U recovery is taken into consideration here. For JSd-2, even the Mw-2 method is sufficient for U recovery (Figure 1). Only ~4.5 h are needed to recover > 90% of U by Mw-2. Hence, compared to Mw-4, Mw-2 is faster and sufficiently effective for U. But to assure recovery of many elements by complete digestion of various reference materials and soil samples (Belarus, Japan, Serbia and Ukraine), Mw-4 was followed as the digestion technique in the present work.

### 2.3. Analysis of REEs, Th and U in Soils from Different Countries

The Mw-4 method was used to digest different soil samples to yield a clear solution. Rare earth elements (REEs), Th and U were analyzed from the solution using ICP-MS. Table 2 gives the concentrations of these elements for the different soils. Errors in the method of analysis are represented as SD based on the number of measurements carried out (*n* = 5). The concentrations of light REEs (LREEs), heavy REEs (HREEs) and total REEs (TREEs) were calculated. The LREE concentration dominates over the HREE concentration in all the soil samples. The LREE/HREE ratios range from 9.36 to 14.41, which indicates high fractionation of REEs in the soils. The ratios are similar for Chernobyl (Ukraine), Gomel (Belarus) and Bratoselce (Serbia) soils. In the case of Japanese soils, the Fukushima soil ratio (9.36) is less than the Hiroshima soil (14.41) ratio. Among the CRMs, JB-1 has a similar ratio to ratios for Chernobyl and Hiroshima soils. The LREE/HREE ratio of JB-3 is the lowest among the CRMs. JSd-2 and JLk-1 values are comparable with that of Fukushima soil. The highest TREE concentration is observed in Bratoselce soil (174.95 μg/g) and the lowest is for Chernobyl soil (51.95 μg/g).

**Figure 3:**
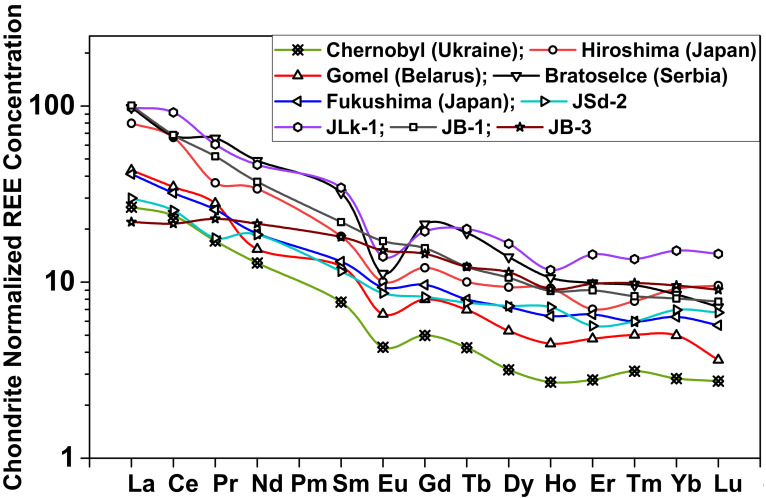
Chondrite-normalized REE patterns of four CRMs and five different soils.

Figure 3 gives the chondrite-normalized REE patterns of the four CRMs and the five different soils collected from Belarus, Japan, Serbia and Ukraine. The patterns are highly fractionated and show different abundances of REEs. The normalized REE patterns show an enrichment in LREE concentration. The HREE concentration patterns are almost flat to depletion with negative Eu anomaly except for the Fukushima soil sample. The fluctuations in concentrations of some of the elements result in deviation from a smooth pattern but it is within the error range of 10%. The degree of accumulation of LREEs with respect to the (La/Sm)_N_ value ranges from 3.05 to 4.38, whereas for the case of HREEs (Gd/Yb)_N_ values are from 1.32 to 2.51 for soil samples.

The Eu anomaly (*Eu_A_*) was calculated using Equation (1), where N stands for normalization with respect to chondrite:

(1) $$Eu_{A}=\frac{Eu_{N}}{\sqrt{Sm_{N}\times Gd_{N}}}$$

The results show that (*Eu_A_*) for the soil samples ranges from 0.43 to 0.83 with an average value of 0.66, indicating the depleted Eu anomaly. The high enrichment in LREEs compared to HREEs with negative Eu anomaly for the soil samples corroborates that their origin may be mainly from the felsic rock types such as granite or granodiorite and clay [25]. The chondrite-normalized REE pattern reveals a natural origin. Therefore, it is difficult to point out any enrichment in REE concentrations in the soil samples from any anthropogenic activity.

Ukraine and Belarus are situated on the Baltic and Ukrainian Shield of the east European craton. The Ukrainian Shield mainly consists of granites, granitoid, schists, sandstone and greenstone belts of rocks. The basement rock types at the soil sample locations of Chernobyl and Gomel are mainly granitic and granodiorite in composition [26]. Serbia is located in southeastern Europe, which is divided into five regional geotectonic units: the Pannonian Basin, Interior Dinarides, Carpatho-Balkanides, Vardar Zone and Serbian-Macedonian Mass. Serbian territory includes a great number of rock complexes. The Šumadija Mountains in central part of Serbia are composed of granitoid and volcanic rock complexes whereas, the Kapaonik Mountains in the southwestern part are composed of granitic rocks, which are concordantly injected into the metamorphic rocks [27]. Hiroshima Prefecture is situated on the granite and granodiorite basement rocks while Fukushima Prefecture is on the granitic basement rocks [28].

The U/Th concentration ratios were calculated for all soils in the present work. The values vary from 0.24 to 0.65 in soils. The soils from Gomel, Fukushima and Hiroshima have ratios comparable to the upper continental crust (UCC) ratio of 0.26, whereas Chernobyl (Ukraine) and Bratoselce (Serbia) soils show high values than the UCC ratio [29]. The maximum concentrations of U (6.4 μg/g) and Th (20.7 μg/g) have been reported in Serbia soils, and they were attributed to the influence of parent rock and accumulation of lithogenic radionuclides; however, their U/Th value was found to be slightly higher (0.31) than the UCC ratio for these soils [27]. Radionuclides of U and Th series are commonly associated in nature and their ratios can be used to estimate pedogenic time frames of soil development or to assess soil processes [30]. The equilibria of the radionuclides are often disturbed by physical and chemical processes that enhance loss or gain of a given decay product. Although the soils from Belarus and Ukraine are typical podzol soils in nature, U enrichment has been observed in Chernobyl soils due to the CNPP accident [31]. The U/Th value infers high concentration of U in soil samples from Chernobyl and Bratoselce in this work, which could be due to significant fractionation during weathering of the soils or any anthropogenic events.

**Figure 4:**
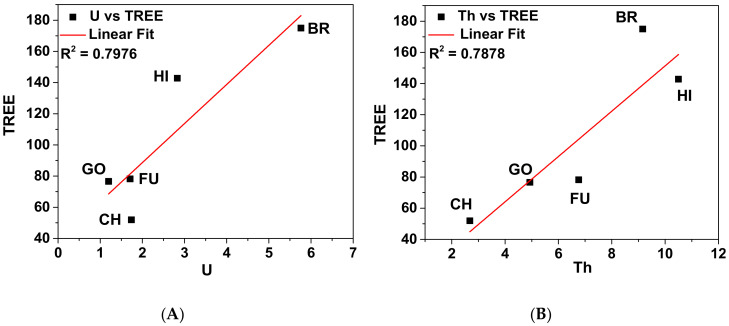
Correlation between (**A**) concentrations of U (μg/g) and TREEs (μg/g) and (**B**) concentrations of Th (μg/g) and TREEs (μg/g) for different soils (BR—Bratoselce, CH—Chernobyl, FU—Fukushima, GO—Gomel, HI—Hiroshima).

Behaviors of U and Th were compared with TREEs for all soils as shown in Figure 4A,B. Figure 4A shows a weak correlation of U with TREEs in Chernobyl, Gomel and Fukushima soils whereas it shows a strong correlation in Bratoselce soil. Figure 4B shows a weak correlation of Th with TREEs in Chernobyl soil whereas it correlates strongly in Bratoselce and Hiroshima soils. A relatively high TREE concentration with a high concentration of Th or U is observed in Bratoselce soil compared to the other soils. This is due to the natural origin of this soil being from granitic rocks. Otherwise, no correlations between Th or U and REEs are observed in soils. The present work could not find any enrichment in REE concentrations in soils except for U enrichment in Chernobyl and Bratoselce soils.

## 3. Materials and Methods

### 3.1. Reagents and Chemicals

All chemical procedures and measurements were performed under clean room conditions (class 10000). All solutions were prepared in de-ionized Millipore water (resistivity = 18.2 MΩ × cm) obtained from a Milli-Q water system (Millipore, Moisheim, France).

Ultrapure analytical grade HNO_3_, HF and HClO_4_ (TAMA-Pure-AA-100, Tama Chemicals Co. Ltd., Kawasaki, Japan) acids were used for soil decomposition. Other chemicals used were of analytical grade.

The geochemical CRMs JSd-2, JLk-1, JB-1 and JB-3 supplied by GSJ (Tsukuba, Japan) were used in the present work.

### 3.2. Samples and Sampling Locations

Surface soil samples were collected at (0–5 cm) depth. A stainless-steel scoop was used to collect about 2 kg of soil from five places randomly distributed within a 100 m^2^ (10 m × 10 m) area. Then these samples were mixed together to form a composite sample. Before collection, grass, litter, roots and shoots were removed from the surface. All soil samples were brought to the laboratory and air dried at room temperature. They were sieved using a 2 mm mesh sieve after manually removing objects like roots, shoots and stones. The sieved samples were oven dried at 110 °C for 24 h. Then, all samples were pulverized using a ball mill to less than 150 μm size prior to chemical decomposition.

Soil samples contaminated with radio cesium activity due to the nuclear accidents at Chernobyl, Fukushima and Gomel were studied in the present work. A soil sample collected from Hiroshima has also been analyzed to extend the analysis of the digestion method. Similarly, a soil sample from an area contaminated with depleted uranium in Bratoselce was analyzed. The details of sampling locations and dates are given in Table 3.

### 3.3. Sample Preparation

All four CRMs were dried in a muffle furnace (Denken Co., Ltd., Kyoto, Japan) at 110 °C for 3 h to remove moisture. The JSd-2 was digested using five different methods, Mw-1, Mw-2, Mw-3, Mw-4 and Sx. Their details are given below.

#### 3.3.1. Microwave Digestion (Mw)

In this method, 0.1 g of dried JSd-2 was transferred separately to closed PTFE vessels for digestion using a microwave digestion system (Milestone Ethos One MA133-003, Sorisole, BG, Italy). Each mixture of concentrated acids was added carefully to avoid drastic reaction.

(A) Mw-1

Microwave digestion was carried out only one time in Mw-1 with a mixture of concentrated acids HNO_3_ (6 mL), HF (2 mL) and HClO_4_ (1 mL).

(B) Mw-2

After Mw-1, mixture of concentrated acids HNO_3_ (2 mL), HF (1 mL) and HClO_4_ (1 mL) was used for the second digestion Mw-2.

(C) Mw-3

For the third digestion Mw-3, mixture of concentrated acids HNO_3_ (1 mL) and HF (1 mL) was added after the successive Mw-1 and Mw-2.

(D) Mw-4

In the fourth digestion Mw-4, the mixture of concentrated acids HNO_3_ (1 mL) and HF (1 mL) was added again. The conditions in the microwave digestion program are given in Table 4. After addition of acid mixtures Mw-1, 2, 3 and 4 the samples were subsequently heated for about 45 min in the microwave digestion system using this program, followed by a cooling cycle for about 60 min or 1 h.

The microwave-digested sample solutions obtained from Mw-1, 2, 3 and 4 methods were transferred to Teflon beakers for evaporation to dryness. Evaporation was continued till removal of any traces of HF and HClO_4_ from the solution. Finally, the residue was dissolved completely in 15 mL of 2% HNO_3_ solution. An experimental blank solution was also processed in a similar way.

#### 3.3.2. Savillex Digestion (Sx)

About 0.1 g of dried JSd-2 was weighed and transferred separately to Savillex PFA vials. A mixture of concentrated acids HNO_3_ (1.5 mL), HF (3.5 mL) and HCl (0.5 mL) was added slowly to avoid drastic reaction. The vials were closed properly and heated for about 12 h at 100 °C. Then the lids were opened and the solutions were dried. Once again, a mixture of concentrated HNO_3_ (3 mL) and HCl (1 mL) was added to closed vials and heated for 12 h at 100 °C. This mixture was also dried. The step of addition of mixture of concentrated HNO_3_ (3 mL) and HCl (1 mL), heating and drying was repeated twice. Finally, the residue was dissolved in 10 mL of 2 M HCl and dried completely. The sample solution was prepared in 20 mL of 2% HNO_3_. An experimental blank solution was also processed in a similar way. The CRMs JLk-1, JB-1 and JB-3 as well as other soil samples were digested with one of the above methods, after deciding the most efficient one. Before digestion, all soil samples were ashed in a muffle furnace (Denken Co. Ltd.) by increasing temperature step-by-step, 100 °C for 2 h, 200 °C for 3 h and 550 °C for 5 h to decompose organic matter from samples.

### 3.4. ICP-MS Measurements

ICP-MS (Agilent 8800, Agilent Technologies, Tokyo, Japan) was used for determination of the concentration of U as well as other elements in soil solution. The operating parameters are given in Table 5. All blanks and samples were diluted using 3% HNO_3_ for ICP-MS measurements. The ICP-MS detection limit was calculated as three times the standard deviation of the calibration blank measurements (*n* = 10). The experimental blanks were prepared similarly. Detection limits varied from 0.03 to 0.2 ng/L for all elements. The calculated detection limit for ^238^U was 0.01 ng/L.

Multi-element Plasma standards solutions of XSTC-1 as well as XSTC-331 (Spex CertiPrep Inc., Metuchen, NJ, USA) spiked with internal standard ^103^Rh were prepared to derive calibration curves. In order to correct the signal attenuation due to the presence of various constituents in the sample solution (known as the “matrix effect”) as well as for possible change in instrumental parameters, an internal standard, ^103^Rh, was used during ICP-MS analysis. The intensities of the abundant stable isotopes of all elements of interest (^139^La, ^140^Ce, ^141^Pr, ^146^Nd, ^147^Sm, ^153^Eu, ^157^Gd, ^159^Tb, ^163^Dy, ^165^Ho, ^166^Er, ^169^Tm, ^172^Yb, ^175^Lu, ^133^Cs, ^88^Sr, ^232^Th and ^238^U) were measured in the present work. From the comparison between the standard and sample intensity ratios, total concentration of that element in the sample was calculated based on its natural abundance of isotopes. The calibration curve was obtained in the analyte concentration range of 0–5000 (ppt or ng/L). The sample solutions were diluted 20-fold for the measurement of REEs, Cs, Th and U whereas 1000-fold for Sr to get a precise working concentration range. Analytical validation of the measurement procedure was carried out using CRMs: JLk-1, JSd-2 (Japanese sediments) and JB-3 (Basalt) supplied by GSJ. The present analysis results were in good agreement with certified values, and their RSD was within 10%.

## 4. Conclusions

Reliable, routine analytical methods for complete digestion of REEs, Cs, Sr, Th and U in geochemical CRMs were successfully carried out using both microwave digestion and Savillex digestion techniques. The efficiency, effectiveness and versatility of Mw-4 were confirmed by the results and it is expected this method will have further applications such as in separation of multi-elements and isotope ratio analysis by mass spectrometry. Microwave digestion method (Mw-2) alone could recover more than 90% U especially, from soil samples within a period of 4–5 h. This method was rapid and more suitable than Savillex digestion. Overall, the present Mw-4 method would be useful for the analysis of multi elements in environmental samples as well as being beneficial in terms of fastness and consumption of less volume of acid thus resulting in an environmentally friendly digestion method.

## Figures and Tables

**Table 1:** Analytical results obtained by ICP-MS for JSd-2, JLk-1, JB-1 and JB-3 in the present study and their comparison with certified values [18].

**Element**	**JSd-2**				**JLk-1**			
	**Mean (μg/g)**	**SD**	**CV (μg/g)**	**Ratio**	**Mean (μg/g)**	**SD**	**CV (μg/g)**	**Ratio**
La	10.56	0.97	11.30	0.93	37.98	3.36	40.60	0.93
Ce	21.68	1.97	23.40	0.93	89.79	1.12	87.90	1.02
Pr	2.43	0.17	2.40	1.01	8.23	0.06	8.53	0.96
Nd	12.19	0.98	13.20	0.92	33.21	0.27	35.70	0.93
Sm	2.55	0.11	2.68	0.95	7.91	0.12	7.87	1.00
Eu	0.82	0.03	0.81	1.01	1.21	0.04	1.27	0.95
Gd	2.67	0.07	2.67	1.00	6.04	0.07	6.02	1.00
Tb	0.43	0.01	0.44	0.98	1.18	0.02	1.23	0.96
Dy	2.76	0.11	2.86	0.97	6.45	0.17	6.57	0.98
Ho	0.64	0.04	0.68	0.94	1.04	0.01	1.06	0.98
Er	1.50	0.13	1.48	1.01	3.66	0.02	3.59	1.01
Tm	0.23	0.01	0.23	1.01	0.52	0.01	0.53	0.99
Yb	1.63	0.06	1.67	0.98	3.76	0.11	3.99	0.94
Lu	0.25	0.01	0.25	0.98	0.56	0.01	0.57	0.99
Cs	1.03	0.03	1.07	0.96	10.51	0.19	10.90	0.96
Sr	200	1	202	0.99	67.32	0.02	67.50	0.99
Th	2.31	0.15	2.33	0.99	18.56	0.96	19.50	0.95
U	1.07	0.03	1.10	0.97	3.75	0.01	3.83	0.98
**Element**	**JB-1**				**JB-3**			
	**Mean (μg/g)**	**SD**	**CV (μg/g)**	**Ratio**	**Mean (μg/g)**	**SD**	**CV (μg/g)**	**Ratio**
La	38.06	0.46	38.60	0.99	8.29	0.53	8.81	0.94
Ce	66.86	0.55	67.80	0.99	20.97	0.15	21.50	0.97
Pr	7.05	0.50	7.01	1.00	3.11	0.04	3.11	1.00
Nd	26.59	0.45	26.80	0.99	15.36	0.32	15.60	0.98
Sm	5.04	0.15	5.13	0.98	4.15	0.22	4.27	0.97
Eu	1.48	0.03	1.49	0.99	1.31	0.04	1.32	0.99
Gd	4.85	0.06	4.90	0.99	4.50	0.10	4.67	0.96
Tb	0.80	0.03	0.82	0.97	0.72	0.02	0.73	0.98
Dy	4.13	0.20	4.14	0.99	4.44	0.22	4.54	0.98
Ho	0.79	0.02	0.79	1.00	0.81	0.03	0.80	1.01
Er	2.29	0.07	2.27	1.00	2.49	0.04	2.49	1.00
Tm	0.32	0.03	0.35	0.91	0.40	0.01	0.42	0.95
Yb	2.01	0.12	2.13	0.94	2.38	0.08	2.55	0.93
Lu	0.30	0.02	0.31	0.97	0.36	0.03	0.39	0.92
Cs	1.24	0.04	1.23	1.00	0.93	0.04	0.94	0.99
Sr	445	1	444	1.00	404	3	403	1.00
Th	9.13	0.06	9.30	0.98	1.23	0.02	1.27	0.97
U	1.60	0.03	1.67	0.96	0.45	0.01	0.48	0.94

Mean = Mean Measured Value; CV = Certified Value; SD = Standard Deviation. Ratio = Mean Measured Value/Certified Value. *n* = 10 for JSd-2. *n* = 5 for JLk-1, JB-1 and JB-3.

**Table 2:** Concentration (± SD) of REEs, Th and U in (μg/g) for different soils. (For, *n* = 5).

Element	Chernobyl, Ukraine	Gomel Oblast, Belarus	Bratoselce, Serbia	Hiroshima, Japan	Fukushima, Japan
La	10.07 ± 0.23	16.33 ± 0.99	37.04 ± 2.55	30.17 ± 1.57	15.53 ± 1.17
Ce	23.31 ± 1.30	33.88 ± 3.21	66.15 ± 5.99	64.78 ± 4.53	31.29 ± 2.28
Pr	2.33 ± 0.21	3.81 ± 0.35	8.92 ± 0.83	4.98 ± 0.37	3.51 ± 0.18
Nd	9.19 ± 0.32	11.02 ± 0.97	35.06 ± 2.91	24.23 ± 2.24	13.50 ± 1.03
Sm	1.77 ± 0.11	2.83 ± 0.25	7.39 ± 0.62	4.19 ± 0.09	3.01 ± 0.14
Eu	0.37 ± 0.02	0.57 ± 0.03	0.97 ± 0.06	0.87 ± 0.04	0.81 ± 0.05
Gd	1.55 ± 0.13	2.48 ± 0.22	6.65 ± 0.61	3.75 ± 0.23	3.01 ± 0.23
Tb	0.25 ± 0.01	0.41 ± 0.04	1.12 ± 0.07	0.59 ± 0.02	0.47 ± 0.02
Dy	1.24 ± 0.05	2.06 ± 0.12	5.42 ± 0.47	3.65 ± 0.27	2.81 ± 0.03
Ho	0.24 ± 0.01	0.40 ± 0.03	0.94 ± 0.05	0.82 ± 0.05	0.57 ± 0.01
Er	0.71 ± 0.03	1.22 ± 0.08	2.52 ± 0.05	1.79 ± 0.16	1.67 ± 0.07
Tm	0.12 ± 0.01	0.19 ± 0.01	0.37 ± 0.01	0.32 ± 0.03	0.23 ± 0.01
Yb	0.70 ± 0.04	1.24 ± 0.05	2.12 ± 0.04	2.28 ± 0.15	1.58 ± 0.11
Lu	0.11 ± 0.01	0.14 ± 0.01	0.28 ± 0.01	0.37 ± 0.02	0.22 ± 0.01
Th	2.68 ± 0.21	4.90 ± 0.21	9.16 ± 0.85	10.21 ± 0.67	6.76 ± 0.58
U	1.74 ± 0.09	1.18 ± 0.07	5.76 ± 0.31	2.73 ± 0.13	1.71 ± 0.08
LREE	48.58	70.92	162.19	132.97	70.65
HREE	3.37	5.66	12.77	9.80	7.55
TREE	51.95	76.58	174.95	142.77	78.20
LREE/HREE	14.41	12.53	12.71	13.57	9.36

**Table 3:** Details of sampling locations and dates.

Sample Name	(Latitude) °N	(Longitude) °E	Date of Sampling
Gomel Oblast, Belarus	52.3922	29.5238	September 1998
Chernobyl, Ukraine	51.3731	29.9949	March 2001
Bratoselce, Serbia	42.3447	21.7560	March 2003
Hiroshima, Japan	34.4742	132.4467	April 2009
Fukushima, Japan	37.5401	140.8672	October 2015

**Table 4:** Details of the microwave digestion program.

Time (min)	Power (W)	T_1_ (°C)	T_2_ (°C)
00:03:00	1000	50	115
00:02:00	0	30	115
00:20:00	1000	210	115
00:20:00	1000	210	115
00:60:00	0	30	30

**Table 5:** Operating parameters for ICP-MS.

**Plasma**	
Frequency (MHz)	27.12
RF Power (kW)	1.55
**Argon Flow (L/min)**	
Plasma	15.0
Carrier	1.0
Nebulizer	Micro Mist type
Sampling distance (mm)	8.0
Sample uptake rate (mL/min)	0.4
**Data Acquisition**	
Mode	Peak jumping mode
Number of points per peak	3
Number of scan sweeps	100
Dwell time per point (s)	0.3
Scan mass range (a.m.u.)	7–238
